# Nuclear factor of activated T-cells, NFATC1, governs FLT3^ITD^-driven hematopoietic stem cell transformation and a poor prognosis in AML

**DOI:** 10.1186/s13045-019-0765-y

**Published:** 2019-07-08

**Authors:** Maria Solovey, Ying Wang, Christian Michel, Klaus H. Metzeler, Tobias Herold, Joachim R. Göthert, Volker Ellenrieder, Elisabeth Hessmann, Stefan Gattenlöhner, Andreas Neubauer, Dinko Pavlinic, Vladimir Benes, Oliver Rupp, Andreas Burchert

**Affiliations:** 1 Department of Hematology, Oncology and Immunology, University Hospital Giessen and Marburg, Campus Marburg, Philipps University Marburg, Marburg, Germany; 2 Laboratory for Leukemia Diagnostics, Department of Medicine III, University Hospital, LMU Munich, Munich, Germany; 3 Department of Hematology, University Hospital Essen, Essen, Germany; 4 Department of Gastroenterology, University Hospital Goettingen, Goettingen, Germany; 5 Institute of Pathology, Justus-Liebig-University, Giessen, Germany; 6 Genomics Core Facility, EMBL Heidelberg , Heidelberg, Germany; 7 Department of Bioinformatics and Systems Biology, University Giessen, Giessen, Germany

**Keywords:** FLT3^ITD^, Acute myeloid leukemia, Hematopoietic stem cells, NFATC1, Drug resistance

## Abstract

**Background:**

Acute myeloid leukemia (AML) patients with a high allelic burden of an internal tandem duplication (*ITD*)-mutated *FMS*-*like Tyrosine Kinase*-*3* (*FLT3*) have a dismal outcome. FLT3^ITD^ triggers the proliferation of the quiescent hematopoietic stem cell (HSC) pool but fails to directly transform HSCs. While the inflammatory transcription factor nuclear factor of activated T-cells 2 (*NFAT2*, *NFATC1*) is overexpressed in AML, it is unknown whether it plays a role in FLT3^ITD^-induced HSC transformation.

**Methods:**

We generated a triple transgenic mouse model, in which tamoxifen-inducible Cre-recombinase targets expression of a constitutively nuclear transcription factor *NFATC1* to *FLT3*^*ITD*^ positive HSC. Emerging genotypes were phenotypically, biochemically, and also transcriptionally characterized using RNA sequencing. We also retrospectively analyzed the overall survival of AML patients with different *NFATC1* expression status.

**Results:**

We find that NFATC1 governs FLT3^ITD^-driven precursor cell expansion and transformation, causing a fully penetrant lethal AML. FLT3^ITD^/NFATC1-AML is re-transplantable in secondary recipients and shows primary resistance to the FLT3^ITD^-kinase inhibitor quizartinib. Mechanistically, NFATC1 rewires FLT3^ITD^-dependent signaling output in HSC, involving augmented K-RAS signaling and a selective de novo recruitment of key HSC-transforming signaling pathways such as the Hedgehog- and WNT/B-Catenin signaling pathways. In human AML, *NFATC1* overexpression is associated with poor overall survival.

**Conclusions:**

NFATC1 expression causes FLT3^ITD^-induced transcriptome changes, which are associated with HSC transformation, quizartinib resistance, and a poor prognosis in AML.

**Electronic supplementary material:**

The online version of this article (10.1186/s13045-019-0765-y) contains supplementary material, which is available to authorized users.

## Introduction

Acute myeloid leukemia (AML) is mainly a fatal disease. AML evolution usually begins with sentinel de novo acquisition of mutations in epigenetic modifier genes such as *DNMT3A*, *ASXL1*, or *TET2*, which also characterize clonal hematopoiesis of healthy individuals, referred to as CHIP or age-related clonal hematopoiesis, ARCH [1]. Progression from CHIP to AML is frequently associated by the consecutive acquisition of additional mutations such as in *NPM1*, *RAS*, *KIT*, or *FLT3* [2]. However, recent evidence also shows that clonal evolution from CHIP to AML is not exactly a linear path [3]. In fact, the requirements, under which CHIP mutations become myeloid driver mutations, cooperate with other genetic or epigenetic changes to eventually cause AML, are not well understood. De novo chromatin alterations that are caused by aberrant transcription factor (TF) signaling could be very critical in this process [4]. One such TF is the nuclear factor of activated T-cells (*NFAT*). *NFATs* are a family of TFs that are physiologically activated by the calcium-activated serine/threonine phosphatase calcineurin, which dephosphorylates NFAT, thereby leading to their nuclear translocation and target gene regulation [5]. NFAT proteins play a pivotal role during T-cell maturation and activation [6]. Aberrant *NFAT* signaling is causally involved in the development of chronic lymphocytic leukemia, non-Hodgkin lymphoma, pancreatic cancer, and several other malignancies. We have previously shown that nuclear NFATC1-induced epigenetic changes convert benign Ras-induced pancreatic adenomas into rapidly lethal, metastatic cancers [7]. NFAT proteins are expressed in normal and malignant lymphatic hematopoiesis. We showed recently that NFATC1 is overexpressed in AML where it mediates resistance to sorafenib in vitro [8]. Sorafenib is a potent inhibitor of the mutated FLT3-receptor tyrosine kinase variant, FLT3^ITD^, which is frequently found in normal karyotype AML [2, 9]. FLT3^ITD^ constitutively activates a signaling network, including the RAS- and STAT-signaling pathways. FLT3^ITD^ confers a poor risk in AML [10, 11]. This provided the rationale for the development of FLT3-specific TKI in order to improve outcome. However, although FLT3-TKI therapy proved to be clinically effective, responses are usually temporary through the development of TKI resistance [12–16]. Notably, although FLT3^ITD^ is a critical driver of AML pathogenesis and dictates prognosis, it fails to transform normal stem cells into self-renewing AML in vivo [17–19] unless other oncogenes are co-expressed [20, 21]. This is because FLT3 ^ITD^-signaling causes overproliferation of normally quiescent stem cells, leading to stem cell depletion rather than leukemic transformation [22].

Here, we show for the first time that NFATC1, a key regulator of inflammatory responses [23], potently synergizes with FLT3^ITD^, by causing stem cell expansion rather stem cell depletion, resulting in the development of a FLT3-kinase inhibitor-resistant lethal AML. In human AML, overexpression of *NFATC1* can be linked to a poor prognosis, supporting the critical importance of NFATC1 in AML biology.

## Results

### Nuclear NFATC1 induces a rapidly lethal FLT3^ITD^-driven leukemia

**Fig. 1:**
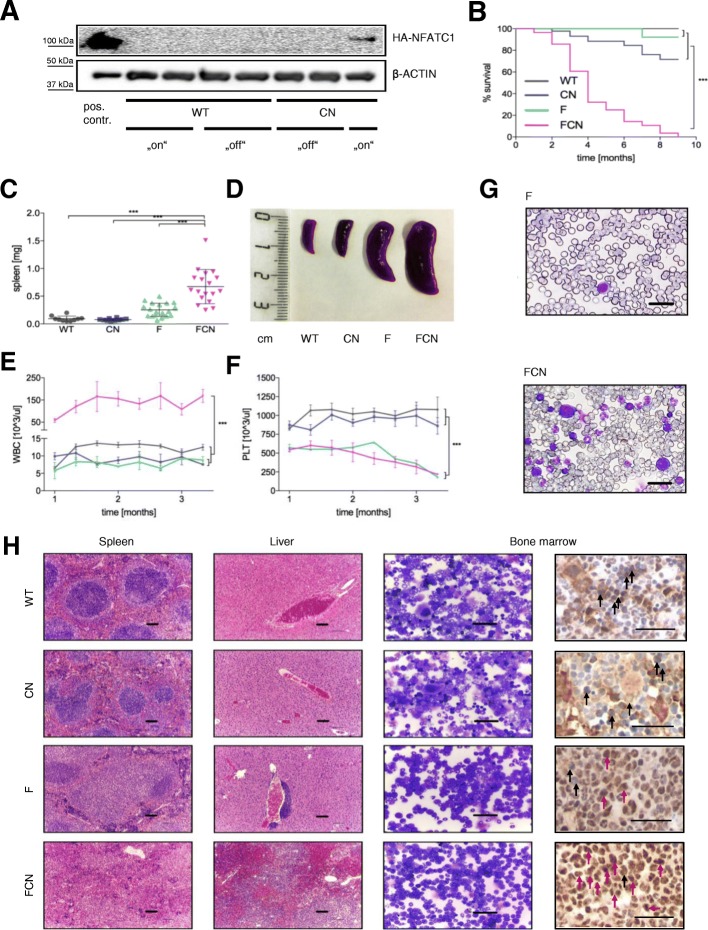
Development of AML in FCN mice. **a** WB of blood samples after erythrocyte lysis before and after induction of NFATc1 expression with tamoxifen treatment of mice. **b** Kaplan Meier survival curve of WT (*n* = 12), CN (*n* = 43), F (*n* = 33), FCN (*n* = 28); *p* < 0.0001. **c**, **d** Spleen weight (mean ± SD, *n* = 10 to 18 per group) (**c**), examples of spleens at 4 months (**d**). **e**, **f** Peripheral white blood counts (**e**) and platelet counts (**f**) over months 1 to 3 (mean ± SEM, *n* = 4 to 10 per group); *p* < 0.001. **g** Peripheral blood smears in F and FCN mice: left shifted hematopoiesis and increased white blood counts in FCN compared to F animals. Scale bars, 50 μm. **h** Bone marrow smears and IHC of bone marrow, histology of spleen and liver of WT, CN, F, and FCN mice. Black arrows highlight brownish cytoplasmic NFATC1 with sparing of blueish nuclear staining. Red arrows indicate nuclear NFATC1. Massive leukemic infiltrates cause a loss of normal tissue structure of spleen and liver in FCN. There are only minor tissue infiltrations in F and normal tissue structure in the CN animals. Bone marrow smears and the bone marrow histology of the FCN mice show increased cellularity, monomorphic infiltrates, as well as restricted erythro- and thrombopoiesis. FCN bone marrow shows stronger colorization and nuclear NFATC1 staining versus cytoplasmic staining patterns in the other genotypes. Scale bars, 100 μm. A Log-rank test (***p < 0.0001) or 1-way ANOVA (***p < 0.001) was used for *p* values

To address whether FLT3^ITD^ synergizes with nuclear NFATC1 during FLT3^ITD^-driven AML transformation, we generated transgenic mice that constitutively express *NFATC1* at the level of hematopoietic stem cells, along with *Flt3*^ITD^ (Additional file 1: Figure S1). To this end, transgenic *NFAT* mice, harboring a hemagglutinin (HA)-tagged constitutively nuclear (c.n.) human *NFATC1* cDNA under the control of a loxP–STOP–loxP cassette in the *ROSA26* locus [7] were crossed with Cre-transgenic mice (*Cre*-*ER*^*T*^ mice [24, 25]), in which tamoxifen-inducible Cre-ER^T^ recombinase is expressed under the control of the stem cell leukemia (*Scl* ) enhancer (SCL-Cre-ER^T^) and transgenic *Flt3*^ITD^ mice [19], thus targeting *NFATC1* expression to *Flt3*^ITD^-positive hematopoietic stem cells. Flt3^ITD^ (F) mice, Scl-Cre;Nfatc1 (CN) mice, and SCL-Cre;Nfatc1;Flt3^ITD^ (FCN) mice were born at the expected Mendelian ratio. F, CN, and FCN mice were viable at birth. Expression of transgenic NFATC1 was confirmed in the blood of animals after tamoxifen induction (Fig. 1a). F mice showed normal survival with only mild myeloproliferative changes characterized by a slight hepatosplenomegaly through white blood cell infiltration (Fig. 1b–h), as reported previously [17–19]. Most CN mice had a normal life expectancy with no bone marrow or organ abnormalities. However, 30% of the CN mice died after a long latency (Fig. 1b) from a leukemia that resembled T-prolymphocytic leukemia (T-PLL) (not shown). In contrast, all FCN mice developed a rapidly lethal myeloid leukemia. Their median survival was only four months and thus significantly shorter than that of F or CN strains (Fig. 1b). Compared to age-matched F, WT, or CN genotypes, FCN mice displayed a significant splenomegaly. The median spleen weight in FCN mice was 0.73 g (range, 0.52–0.83) and thus significantly higher than in F animals (median 0.2 g; range, 0.19–0.31) (Fig. 1c, d). When compared to F, WT, or CN mice, FCN mice also displayed a dramatically increased leukocytosis. When compared to CN and WT animals, F and FCN animals displayed thrombocytopenia and anemia (Fig. 1e–g). While bone marrow, spleen, and liver histologies of CN mice were unremarkable, F animals showed myeloproliferative changes in bone marrow and spleen, but only a mild leukemic liver infiltration (Fig. 1h). In contrast, bone marrow of FCN animals was characterized by a massively expanded immature hematopoiesis, monocytic infiltrates and suppressed erythro- and thrombopoiesis, destroying the physiological organ structure (Fig. 1h). Immunohistochemistry demonstrates that leukemic infiltrates in FCN animals show characteristic nuclear localization of NFATC1 (Fig. 1h). Together, constitutively active NFATc1 critically augments FLT3^ITD^-driven leukemogenesis.

### NFATC1 increases FLT3^ITD^-dependent clonogenic cell mass through precursor and stem cell expansion

**Fig. 2:**
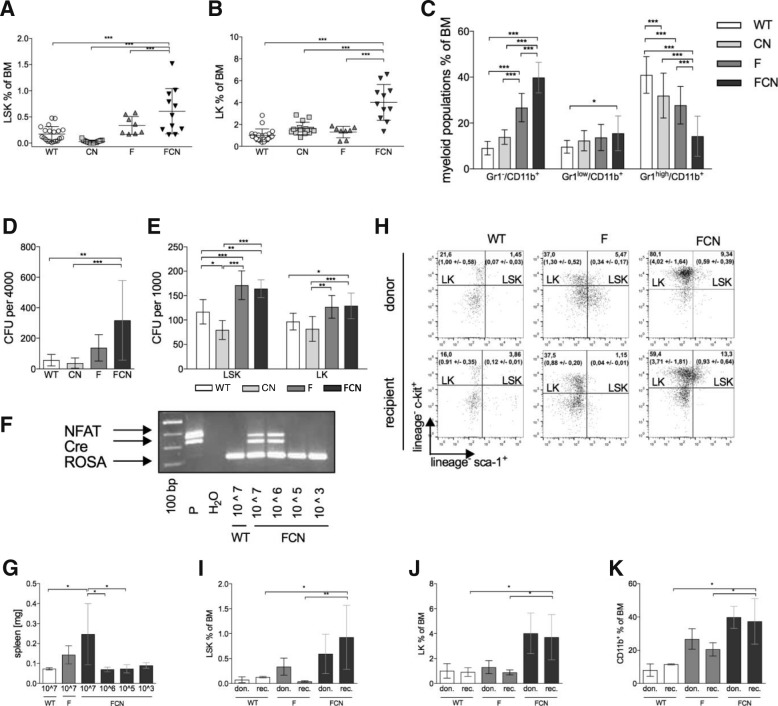
Immunophenotype and transplantability of AML in FCN mice. (**a**–**c**) Immunophenotype of bone marrow. Relative frequency of LSK (**a**), LK (**b**), and myeloid populations (**c**) in total bone marrow (mean ± SD. *n* = 8 to 20). (**d**–**e**) CFU number from total bone marrow (**d**) or sorted LSK and LK (**e**) (mean ± SD. *n* = 3 to 9). **f**–**k** Analysis of mice transplanted with total bone marrow cells at different cell concentrations. Genotyping-PCR to assess leukemic engraftment depending on the total number of transplanted cells (**f**). Spleen weight of recipient animals as a function of the number of transplanted primary leukemia cells (**g**). Representative comparison of the frequencies of stem cells (LSK) and progenitor cells (LK) in leukemic donor and recipient mice, which had received 10^7^ total donor cells (**h**). Proportions of LSK (**i**), LK (**j**), and CD11b^+^ (**k**) in the bone marrow of recipient mice, which have received 10^7^ total donor cells (mean ± SD. *n* = 3 to 4). A one-way ANOVA or 2-way ANOVA with Bonferroni multiple comparison (**a**–**e**) or Dunnett’s post-test (**g**, **i**–**k**) was used to assess the significance of differences (*p* values: *p < 0.05, **p < 0.01, ***p < 0.001.)

Immunophenotypic analysis of FCN leukemia from bone marrow (Fig. 2a–c) and spleen (Additional file 2: Figure S2) of WT, F, CN, and FCN animals was performed. While constitutive NFATC1 signaling alone (CN mice) causes bone marrow hypo-cellularity with a marked reduction of Lin^neg^Sca1^pos^c-kit^pos^ (LSK) stem cells when compared to all other genotypes (Fig. 2a), co-expression of FLT3^ITD^ with NFATC1 in FCN mice leads to a significant expansion of the LSK- and Lin^neg^c-kit^pos^ (LK) compartments in bone marrow (Fig. 2a, b) and the spleen (Additional file 2: Figure S2A, B), suggesting that cooperativity between FLT3^ITD^ and NFATC1 causes stem and progenitor cell expansion. Of note, LK progenitors in the bone marrow of FCN mice are predominantly granulocyte-monocyte progenitors (GMP) (Additional file 2: Figure S2C). While having strongly reduced mature granulocytes (Gr-1^high^/CD11b^+^) (Fig. 2c, Additional file 2: Figure S2D) compared to F and CN mice, FCN mice display a profoundly increased monocytic population (Gr-1^−^/CD11b^+^) in bone marrow and spleen. NFATC1/FLT3^ITD^ cooperatively mediate myeloid precursor and stem cell expansion at the expense of maturation of B220^+^ B-cell, CD3^+^ T-cell, and TER119^+^/CD41^−^ erythroid lineages (Additional file 2: Figure S2E, F). To assess, whether FLT3^ITD^ and NFATC1 signaling cooperativity alters colony formation capacity, the CFU potential of total marrow cells, stem and progenitor cell populations, from WT, CN, F, and FCN mice was compared in vitro. While total bone marrow (Fig. 2d) and sorted LSK and LK populations (Fig. 2e) of F or FCN mice contained more clones with CFU potential than WT or CN mice, the highest CFU counts were detected in FCN mice (Fig. 2d). Thus, increased CFU counts in FCN mice were a consequence of the increase of total LSK and LK and increasing colony forming capability within these compartments (Fig. 2a, b). Intriguingly, NFATC1-signaling alone significantly reduced the fraction of LSK with CFU potential compared to WT, F, and FCN mice (Fig. 2e). Thus, although NFATC1 signaling alone inhibits colony formation capacity of LSK, contemporary FLT3^ITD^ signaling dramatically overcomes this effect.

FCN leukemias were transplantable. When 10^6^ and 10^7^ FCN leukemia cells were injected into sub-lethally irradiated WT mice, which were sacrificed after 3 months of observation. FCN engraftment was seen in 1/3 and 3/3 animals, respectively (Fig. 2f), while no engraftment was seen in mice that received 10^3^ and 10^5^ cells. Recipient animals developed a monocytic leukemia and splenomegaly (Fig. 2e). Importantly, in contrast to secondary F leukemias, which showed less LSK and LK than primary F leukemias (Fig. 2h, i), as exemplary shown (Additional file 3: Figure S3), secondary recipients of FCN leukemias fully re-established the FCN leukemia phenotype, maintained or even increased the proportions of LSK, LK, and monocytic fractions (Fig. 2h–k).

### NFATC1 causes primary resistance to the FLT3-inhibitor quizartinib

**Fig. 3:**
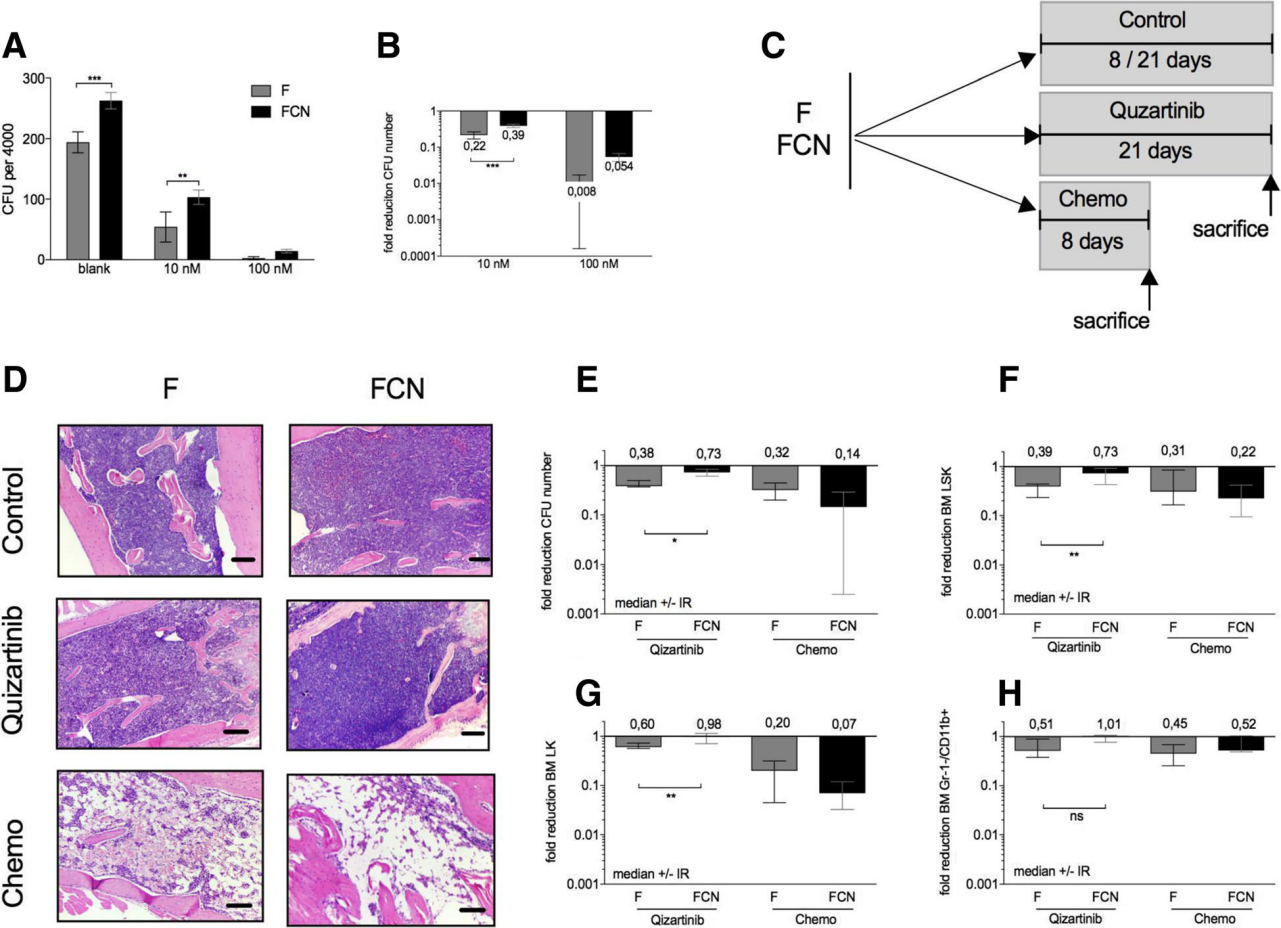
Analysis of treatment sensitivity to quizartinib and chemotherapy. (**a**, **b**) Response to in vitro cytarabine treatment of total bone marrow. CFU number after treatment (**a**). Normalized CFU reduction (**b**). (mean ± SD. *n* = 3) 2-way ANOVA with Bonferroni multiple comparison was used for *p* values. **c** Scheme of in vivo treatment. **d** Histology of the bone marrow of AC220- and chemotherapy-treated groups. Scale bar, 100 μm. (**e**–**h**) Normalized CFU number reduction (**e**), normalized bone marrow LSK reduction (**f**), normalized bone marrow LK reduction (**g**), normalized bone marrow Gr-1^−^/CD11b^+^ reduction (**h**) (median ± IR. *n* = 6 to 10). Mann-Whitney test was used for *p* values. *p < 0.05, **p < 0.01, ***p < 0.001

Clinical efficacy of FLT3^ITD^-specific TKI in the relapsed or refractory situation is often limited by the development of resistance [13, 15, 16]. We have previously shown that NFATC1 mediates sorafenib resistance in vitro. Here, we asked, whether increased NFATC1 expression causes resistance to quizartinib [26], a more potent FLT3^ITD^ inhibitor, or to standard chemotherapy in primary F and FCN leukemia cells in vitro and in vivo. After in vitro treatment with cytarabine at 10 nM and 100 nM, FCN bone marrow formed significantly more colonies in soft agar compared to F bone marrow, suggesting NFATC1 induces cytarabine resistance (Fig. 3a, b). We also investigated the sensitivity of F and FCN leukemia to quizartinib vs. chemotherapy in vivo. To this end, three cohorts, respectively, of FCN and F mice (average age of 6 months) were treated with doxorubicin plus cytarabine (5 + 3 days), treated with quizartinib (21 days), or mock treated with vehicle only (Fig. 3c). Mice were sacrificed the day following last treatment, and bone marrow and spleens were harvested. At this time, bone marrow histology showed a slight reduction in marrow cellularity through quizartinib in F but not in FCN mice, and a stronger cytopenia after chemotherapy in F and FCN mice (Fig. 3d). Moreover, while F mice showed an up to 61% reduction in CFU counts, LSK, and LK numbers after quizartinib treatment, FCN animals did not (Fig. 3e–g), suggesting NFATC1-induced quizartinib resistance. NFATC1-induced cytarabine resistance in vitro could be overcome by combination chemotherapy in vivo (Fig. 3e–h). Of note, monocytic cells (Gr-1^−^/CD11b^+^) were more resistant to chemotherapy or quizartinib than stem and progenitor cells (Fig. 3h).

### De novo recruitment of NFATC1 dependent leukemic signaling pathways in the context of FLT3^ITD^

**Fig. 4.**
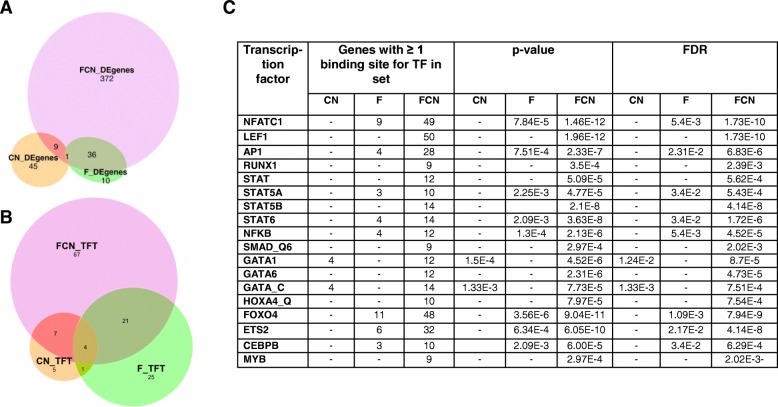
RNA sequencing of stem cells. **a** Euler diagram of DE genes with Log2FC greater 1 or less − 1 and FDR < 0.01. **b** Euler diagram for analysis from GSEA C3 TFT database. **c** Summary table for analysis from GSEA C3 TFT database

**Fig. 5.**
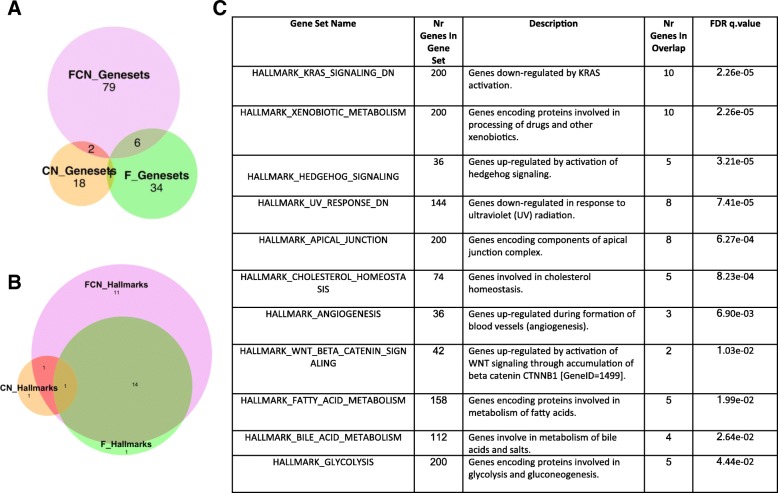
GSEA analysis from C3 TFT database. **a** Euler diagram for analysis from GSEA C2 database. **b** Euler diagram for analysis from GSEA H database. **c** Summary table for analysis from GSEA H database: 11 pathways exclusively enriched in FCN

To obtain insights into the transcriptional deregulations that underlie phenotypic changes of FCN mice, we compared the transcriptome of hematopoietic stem cells (LSK) from FCN, F, and CN mice to WT mice using RNA sequencing. This revealed that FCN-LSK express substantially more differentially expressed (DE) genes than F or CN LSK (Fig. 4a). Furthermore, data support that constitutively nuclear NFATC1 causes a profound qualitative gene expression shift in the context of FLT3^ITD^ signaling, because only a minority of DE genes in FCN (*n* = 417) overlaps with DE genes in F alone (*n* = 36) (Fig. 4a). Most of the DE genes of CN stem cells (*n* = 54) did not overlap with FCN DE genes. Most of the DE genes in FCN stem cells encode for membrane proteins, followed by DE genes encoding for secreted, cytoplasmatic, and nuclear proteins (data not shown). Gene set enrichment analysis (GSEA) analysis for transcription factor targets (TFT) revealed 17, 51, and 99 candidate TFs with at least one binding site in the significantly regulated DE gene sets of CN, F, and FCN stem cells, respectively (Fig. 4b). This implies that NFATC1 enables transcriptional activation in stem cells exclusively in the context of FLT3^ITD^ expression. Intriguingly, TFs, which—according to their predicted binding sites in DE genes—only bound to their target genes in FCN stem cells such as *LEF1*, *RUNX1*, *STAT5B* were previously shown to be critically involved in leukemic HSC transformation, especially also FLT3^ITD^-induced transformation [20, 27, 28] (Fig. 4c). Using gene set enrichment analysis, we investigated the involvement of F, CN, and FCN DE genes in the regulation of different biological processes, reflected by different C2 gene sets. Among the 87 significantly regulated C2 gene sets in FCN stem cells, only 6 are also deregulated by F stem cells, suggesting that NFATC1 critically governs recruitment of genes into novel gene sets in an FLT3^ITD^-dependent manner (Fig. 5a). GSEA of the involvement of FCN DE genes in hallmark cancer pathways uncovered the K-RAS-, Hedgehog-, and WNT/B-Catenin-signaling pathways as well as glycolysis signaling among the 11 significantly activated oncogenic pathways that were specifically recruited through the cooperation of NFATC1 and FLT3^ITD^ in FCN stem cells (Fig. 5b, c). There were further 14 oncogenic hallmark pathways that were significantly activated both in F and especially in FCN HSC, among them inflammation-, TP53-, TNF-alpha-, and JAK-STAT signaling and epithelial-mesenchymal transition, a process known to be regulated by NFAT in embryonic stem cells (Additional file 4: Figure S4).

### NFATC1 and FLT3-signaling govern poor survival in AML

**Fig. 6.**
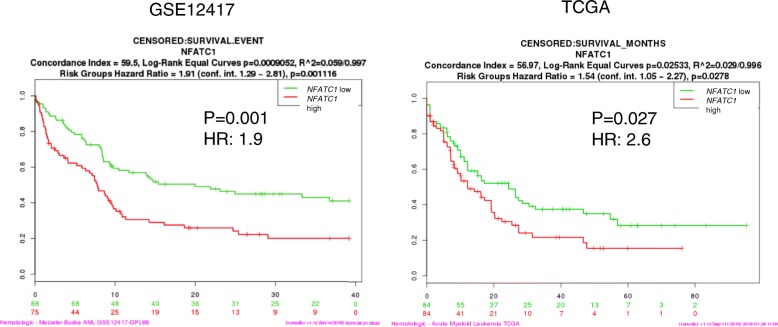
Overall survival in AML patients according to high or low *NFATC1* expression levels. KM plots were generated using a median split

We studied, whether *NFATC1* expression is of prognostic importance in AML. Survival of two independent AML cohorts, GSE12417 [29] (*n* = 163) and TCGA [30] (*n* = 168), was analyzed according to high or low *NFATC1* expression levels using the SurvExpress online biomarker validation tool [31]. In both AML cohorts, *NFATC1*- overexpression was associated with a significantly worse overall survival (Fig. 6a, b).

## Discussion

Here, we provide evidence that NFATC1 is an essential driver of FLT3^ITD^-dependent leukemic transformation and demonstrate a previously unknown prognostic role for *NFATC1* in AML. By generating a transgenic *Flt3*^ITD^-mouse model with tamoxifen-inducible targeted expression of *NFATC1* in HSC, we show that expression of nuclear NFATC1 is a key factor driving FLT3^ITD^-induced stem cell expansion and transformation and confers primary FLT3^ITD^ inhibitor resistance.

Development of this mouse model was prompted by our recent observations showing that many *FLT3*^ITD^-positive and -negative AML patients frequently overexpress *NFATC1*. We have also shown previously in a murine model of inflammation-driven pancreatic cancerogenesis that NFATC1 is a relay switch converting *Kras*^G12D^-driven pre-neoplastic changes into a rapidly lethal metastatic pancreatic cancer [7]. Here, we demonstrate that expression of the nuclear NFATC1 in stem cells changes the biological outcome of aberrant FLT3^ITD^ kinase signaling. In the presence of NFATC1, FLT3^ITD^-signaling leads to extensive precursor cell expansion, development of a fully penetrant lethal and quizartinib-resistant AML. In contrast, neither expression of FLT3^ITD^ nor NFATC1 alone in HSC causes an apparent phenotype in vivo.

Fundamental gene expression changes underlie the biological differences among F, CN, and FCN animals. A GSEA-TFT-based alignment of differential gene expression patterns and target motifs for TFs in the promoters/enhancers of DE genes strongly suggested that there is qualitative and quantitative cooperativity between NFATC1 and FLT3^ITD^. Qualitative cooperativity is presumably due to an NFATC1-dependent de novo recruitment of TFs to promoters in FCN, but not F or CN DE genes. Among them were TFs that are known to be involved in stem cell transformation such as LEF1 [32], HOXA4 [33] or FLT3^ITD^-dependent AML transformation, such as RUNX1 [20], STAT5 [28, 34], and MYB [35]. In line with these qualitative changes in gene expression, key self-renewal pathways of leukemogenesis, such as the Hedgehog- and WNT/B-Catenin signaling pathways, are recruited de novo only in FCN stem cells. Both these pathways were shown to be essential for the maintenance of cancer stem cells in myeloid leukemias and can be specifically targeted by therapeutic intervention [36, 37].

According to our GSEA TFT analysis, NFATC1 enables the recruitment of FLT3^ITD^-inducible TFs such as the MAPK-inducible TFs AP1 and NF-κB, STAT5 [34], and FOXO4 [38] to significantly more target genes than would be accessible with FLT3^ITD^ expression alone. RNA sequencing indeed suggests a dramatic amplification of FLT3^ITD^-signaling output in the presence of nuclear NFATC1, that is, recruitment of more genes into existing FLT3^ITD^-dependent gene sets. Intriguingly, GSEA-TFT analysis of FCN stem cells shows activation of known NFATC1 binding partners AP1 and RUNX1 [39, 40], which were recently uncovered as key drivers of an open chromatin structure in FLT3^ITD^-positive primary AML blasts [41]. This supports that NFATC1’s transforming potential in the context of oncogenic FLT3^ITD^-signaling is linked to its capability to induce or promote active chromatin states [7, 39, 42].

Chromatin-modifying roles of TFs are not unprecedented. They have been reported for TF involved in leukemogenesis such as TCF/LEF1 [37] or FOX [43]. Our data suggest that NFATC1/FLT3^ITD^ cooperativity leads to a gain of transcriptional plasticity, which is an important prerequisite for the acquisition of increased fitness and thus development of phenotypic diversity such as drug resistance under selective pressure [44] (Additional file 5: Figure S5).

In contrast to F animals, FCN animals were resistant to a clinically effective FLT3^ITD^-inhibitor, quizartinib [45], because only F animals showed a significant reduction of LK and LSK. The fact that quizartinib did not more potently eliminate F leukemia might be explained by the fact that in contrast to human FLT3^ITD^ positive AML, FLT3^ITD^ fails to induce oncogenic dependence in FLT3^ITD^-induced myeloproliferation in F mice. In AML patients, FLT3^ITD^ induces oncogene dependence as the basis of therapy response to FLT3 inhibition [16], yet remissions are short lived and mostly not curative [13, 15, 46]. We have previously shown that NFATC1-induced FLT3^ITD^-inhibitor resistance is FLT3^ITD^-independent [8]. In contrast to FLT3^ITD^, bromodomain and extra-terminal (BET) proteins, such as BRD4, which read chromatin and regulate transcription, are non-oncogene addiction targets in MLL-AF9-positive AML [47]. BET inhibitor resistance is mediated via activation of WNT signaling, which involves a profound transcriptional reprogramming [48]. Thus, it is tempting to speculate that WNT-signaling activation through FLT3^ITD^/NFATC1 cooperativity (Fig. 5d) mediates FLT3^ITD^-independent FLT3-inhibitor resistance that could be overcome using BET- or WNT-signaling inhibitors.

Moreover, the recruitment of independent oncogenic signaling pathways through FLT3^ITD^/NFATC1 cooperativity might explain, why oncogenic driver mutations such as FLT3^ITD^—though being instrumental for AML initiation—become dispensable for AML maintenance, once NFATC1 is overexpressed. The importance of NFATC1 overexpression for AML transformation as suggested by the FCN mouse model is supported by the observation of a dismal prognostic influence of *NFATC1* overexpression in AML. Ongoing studies will reveal whether pre-emptive blocking of inflammatory signals that trigger NFATC1 expression or NFATC1-activation itself using cyclosporine could be a concept to prevent NFATC1-dependent leukemogenesis.

## Methods

### Transgenic animals

*Flt3*^*ITD*^ mice (B6.129 background) were bought from Jackson Laboratory, transgenic *NFATC1* mice and conditional Cre-SCL-HSC-ER mice (both C57Bl/6j background) generated as described previously [7, 24] (for breeding scheme, see Additional file 1: Figure S1). All mice (except recipient mice in transplantation experiment) were treated with CreActive TAM400 (LASvendi LASCRdiet) tamoxifen diet for 21 days. All animal procedures were conducted in accordance with TierSchG and were approved by RP Giessen.

### Blood analysis

A total of 50 μl of blood was taken from the tail vein and max of 10% (*v*/*v*) of Di-Sodium-EDTA 1.107% solution was added to prevent coagulation. Blood films were stained with Wright-Giemsa method. For blood cell counts, a dilution of 1:7 in NaCl 0.9% was made and measured in a capillary mode on SYSMEX XS 1000i.

### PCR

See Additional file 6: Table S1 for primer and PCR reaction information.

### Cytospins and pathology

Cytospins were prepared with 2 × 10^5^ cells and stained with Wright-Giemsa. For pathology, mouse organs were fixed in 4% PFA (paraformaldehyde) and stained with H&E.

### Western blotting

Blood samples for Western blotting were obtained from the tail vein and erythrocytes were lysed. Total protein lysates and transfer were performed as described previously [19]. The following primary antibodies were used: mouse monoclonal anti-HA, clone 6E2 (Cell Signaling, Danvers, USA), mouse monoclonal anti-beta-actin, and clone AC-15 (Sigma-Aldrich, Steinheim, Germany). Secondary goat anti-mouse horseradish peroxidase (HSP)-conjugated antibody was used (Dako Cytomation, Glostrup, Denmark) (see Additional file 7: Table S2).

### Immunohistochemistry

Sections of paraffin-embedded bone marrow were immunohistochemically stained using the NovolinkTM Polymer Detection Kit (Leica, Wetzlar, Germany), pH 6.0 for antigen unmasking and the NFAT2 monoclonal antibody, clone ab25916 (Abcam, Cambridge, MA, USA) (see Additional file 7: Table S2).

### Flow cytometry and cell sorting

Flow cytometry analysis was performed as previously described [28]. Cell sorting was performed using the same antibodies and staining protocols (for the list of antibodies used, see Additional file 7: Table S2).

### In vitro colony forming assays

Total bone marrow after erythrocyte lysis or sorted populations were seeded into full cytokine methylcellulose medium (Methocult M3434, STEMCELL Technologies) at 4000 cells for total bone marrow or 500 cells for sorted LSK or LK per well, respectively. Colonies were counted at 7 to 14 days. Cells were re-plated at the same cell numbers.

### Bone marrow transplantation

Total bone marrow from diseased animals or WT controls was isolated, erythrocytes were lysed and four different concentrations of total cells (1 × 10^7^, 1 × 10^6^, 1 × 10^5^, and 1 × 10^3^) transplanted via tail vein injection into sub-lethally (7 Gy) irradiated WT recipients. According to the mouse experimental proposal, transplanted recipient mice were sacrificed and analyzed 3 months after transplantation.

### In vitro and in vivo treatment

For in vitro cytarabine treatment, total bone marrow cells were seeded after erythrocyte lysis into 6-well plates at 4 × 10^4^ cells per well in Iscove’s Modified Dulbecco’s Medium (plus 20% fetal calf serum, 2% penicillin/streptomycin and cytokines: IL-3, IL-6, stem cell factor 0.1%) and treated with 0.9% sodium chloride solution or cytarabine at concentrations of 10 nM or 100 nM for 24 h. Cells were washed with phosphate-buffered saline and seeded into methylcellulose medium in triplicates at 4 × 10^3^ cells per dish (as described [8, 49]). The colony number was evaluated after 7–10 days.

For in vivo treatment, the AC220 treatment group received AC220 daily at 10 mg/kg, suspended in 5% 2-hydroxypropyl-ß-cyclodextrin for 21 days. The chemotherapy group received intraperitoneal injections of 100 mg/kg cytarabine for 5 days and 3 mg/kg doxorubicin for 3 days. Both control groups received solvent.

### RNA sequencing and analysis

Total RNAs were isolated from sorted mouse bone marrow stem cells (LSK, *n* = 2 to 3 per genotype) using RNeasy Micro Kit (Qiagen), according to the manufacturer’s protocol. For WT and CN genotypes, several bone marrow samples were pooled together to get enough material per one mRNA sample. RNA libraries were generated with the NEBNext ® UltraTM RNA Library Prep Kit for Illumina (New England Biolabs® Inc.), according to the protocol of the manufacturer. The libraries were sequenced on an Illumina HiSeq 2000, requested Read Length HiSeq 2 × 75 bp with paired end.

Mapping was done with bwa aligner to GRCm38 (mm10) mouse genome (see Additional file 8: Table S3 for raw counts). DESeq2 was used to calculate differentially expressed genes. DE genes were filtered by FDR adjusted *p* value less than 0.01 and log2 fold change greater than 1 and less than − 1 (see Additional file 9: Table S4 for DE genes). The information on the subcellular localization of the proteins coded by the DE genes was collected from the databases http://www.uniprot.org and https://imb.uq.edu.au. GSEA analysis [50] was performed with the filtered DE genes on the following platform: http://software.broadinstitute.org/gsea/msigdb/annotate.jsp. We used H, C3 TFT, or C2 MSigDB databases and considered only gene sets with FDR *q* value less than 0.01.

### AML patient data analysis

Overall survival of AML patients was analyzed according to *NFATC1* expression levels. Kaplan Meier plots were generated for the data sets GSE21417 and TCGA using SurvExpress—a biomarker validation tool and database for cancer gene expression data [31]. The median *NFATC1* expression level was used as the threshold to split AML patients into two high versus low *NFATC1*-expressing cohorts.

### Statistical analysis

Data, which followed normal distribution, are presented as mean ± SD. Differences between groups were analyzed by one-way ANOVA or two-way ANOVA with Bonferroni or Dunnett’s multiple comparison post testing; *p* < 0.05 was considered significant. The data, which did not follow normal distribution, are presented as median ± IR. The differences between groups were analyzed by Mann-Whitney non-parametric test. Log-rank test was used to calculate the *p* value of the difference in the OS.

## Conclusions

For the first time, it is shown that NFATC1—a transcription factor that is induced upon inflammatory stimuli—converts FLT3^ITD^-induced myeloproliferation into an FLT3^ITD^ inhibitor-resistant, lethal AML, which is associated with NFATC1-dependent de novo recruitment of key oncogenic signaling pathways. This implies that therapeutic targeting of these pathways, NFATc1 itself using calcineurin inhibitors or inflammation could be a strategy to treat NFATc1-expressing myeloid neoplasias.

## Additional files

Additional file 1: **Figure S1.** Breeding scheme. The genotypes used for the experiments are marked with red boxes: *FLT3*^*ITD*^ homozygous (F+/+), *SCL*-*HSC*-*Cre*^*ER*^:*NFATC1* homozygous (CN+/+), and *FLT3*^*ITD*^:*SCL*-*HSC*-*Cre*^*ER*^:*NFATC1* homozygous (F+/+CN+/+). Note: in all the *SCL*-*HSC*-*Cre*^*ER*^–baring animals, the zygosity of the *Cre* allele is unknown. (PPTX 44 kb)

Additional file 2: **Figure S2.** Immunophenotyping of bone marrow and spleen. (A) Relative frequencies of LSK (mean ± SD. *n* = 6 to 16). (B) Relative frequencies of LK (mean ± SD. *n* = 6 to 16). (C) Relative frequencies of GMP, CMP, and MEP (mean ± SEM. *n* = 3 to 4). (D) Relative myeloid populations (mean ± SD. *n* = 6 to 16). (E) Relative frequencies of B-cells (B220^+^), T-cells (CD3^+^), erythrocytes (TER119^+^) and megakaryocytes (CD41^+^) (mean ± SEM. *n* = 3 to 10). (F) Relative frequencies of B-cells (B220^+^), T-cells (CD3^+^), erythrocytes (TER119^+^) and megakaryocytes (CD41^+^) (mean ± SEM. *n* = 3 to 10). A 1-way ANOVA or 2-way ANOVA with Tukey’s multiple comparison was used for *p* values. *p < 0.05, **p < 0.01, ***p < 0.001. (PPTX 1112 kb)

Additional file 3: **Figure S3.** Immunophenotyping of bone marrow and spleen after transplantation into CN or WT recipients. 10^7^ total bone marrow cells from F or FCN donor mice were transplanted into irradiated WT recipients. FACS analysis of LSK/LK and myeloid populations are shown for each recipient mouse. (PPTX 484 kb)

Additional file 4: **Figure S4.** GSEA analysis from C3 H database for mutual FCN and F genes. Summary table for analysis from GSEA H database: 14 pathways enriched in both FCN and F (only values for FCN are shown). (PPTX 80 kb)

Additional file 5: **Figure S5.** Working model. Activation of NFATC1 by an inflammatory environment rewires the transcriptional program induced by FLT3-ITD in stem cells, which promotes leukemic transformation. The FLT3-ITD/NFATC1 synergism activates multiple oncogenic pathways such as WNT/B-Catenin, Hedgehog, and K-RAS, causing extensive proliferation and drug resistance. (PPTX 91 kb)

Additional file 6: **Table S1.** PCR primer. (XLSX 36 kb)

Additional file 7: **Table S2.** Antibodies. (XLSX 42 kb)

Additional file 8: **Table S3.** Counts. (XLSX 898 kb)

Additional file 9: **Table S4.** DE genes. (XLSX 4937 kb)

## Data Availability

The datasets used and/or analysed during the current study are available from the corresponding author on reasonable request.

## Abbreviations

AML: Acute myeloid leukemia
ARCH: Age-related clonal hematopoiesis
BET: Bromodomain and extra-terminal protein
CFU: Colony forming units
CHIP: Clonal hematopoiesis of indetermined potential
CMP: Common myeloid progenitor
CN: Double transgenic *Scl*-*HSC*-*Cre*-*ER*^*T*^:*NFATC1* mouse
DE genes: Differentially expressed genes
ER^T^: Estrogen receptor, induced by tamoxifen
F: Transgenic *Flt3*^*ITD*^ mouse
FCN: Triple transgenic *Flt3*^*ITD*^:*Scl*-*HSC*-*Cre*-*ER*^*T*^:*NFATC1* mouse
GMP: Granulocyte-monocyte progenitors
GSEA: Gene set enrichment analysis
HA: Hemagglutinin
HSC: Hematopoietic stem cells
LK: Mouse Lin^neg^c-kit^pos^ progenitor cells
LSK: Mouse Lin^neg^Sca1^pos^c-kit^pos^ stem cells
MEP: Megakariocate-erythrocyte progenitor
OS: Overall survival
TCGA: The cancer genome atlas project
TF: Transcription factor
TFT: Transcription factor targets
TKI: Tyrosine kinase inhibitor
T-PLL: T-prolymphocytic leukemia
WT: Wild type mouse
