# Supplementary figures and images for: Nuclear factor of activated T-cells, NFATC1, governs FLT3ITD-driven hematopoietic stem cell transformation and a poor prognosis in AML
Source: J Hematol Oncol. 2019 Jul 8;12:72. doi: 10.1186/s13045-019-0765-y (PMC6615262; doi:10.1186/s13045-019-0765-y)

## Slide 1
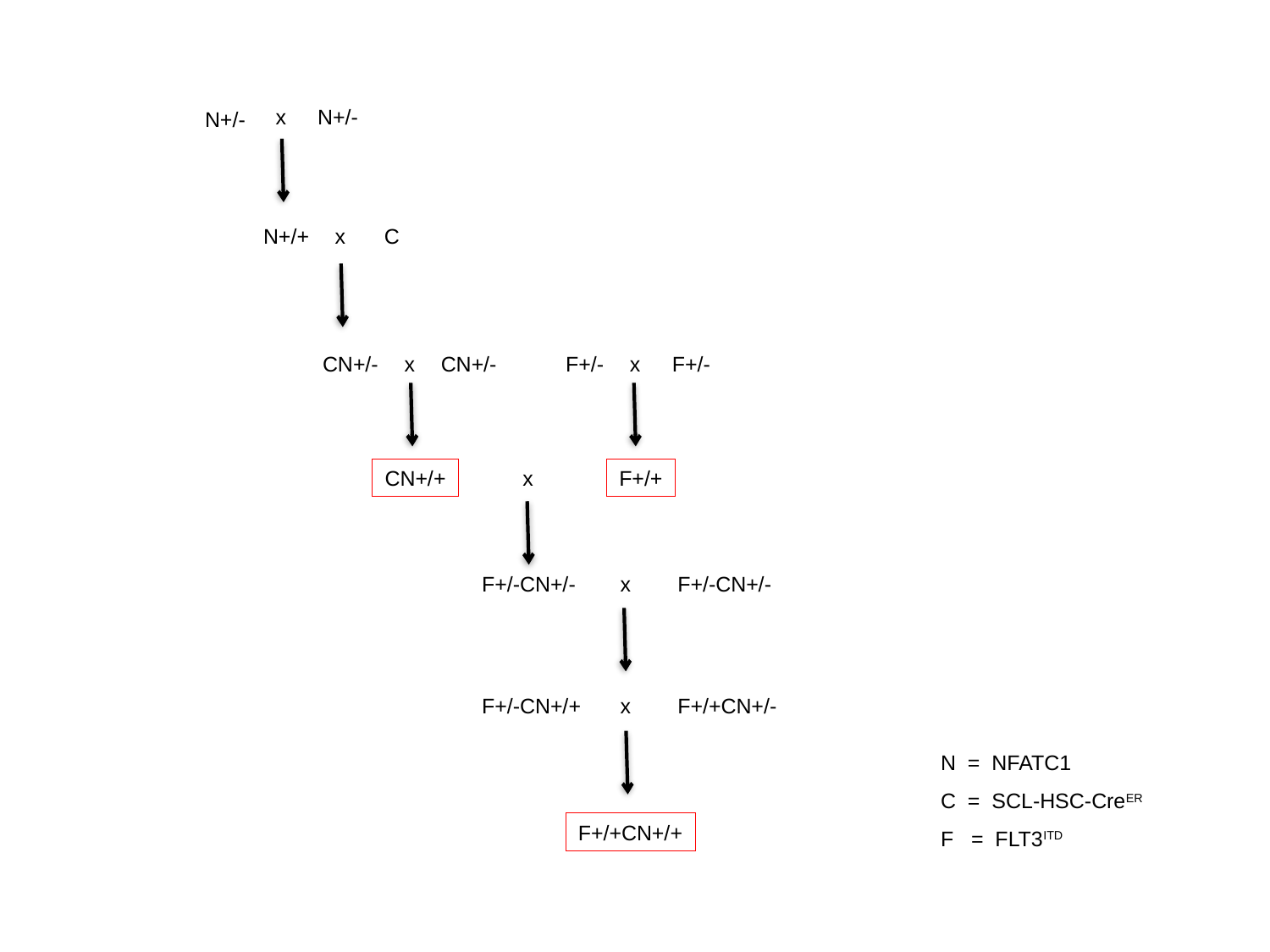

x
N+/-
N+/-
N+/+
x
C
CN+/-
x
CN+/-
F+/-
x
F+/-
CN+/+
x
F+/+
F+/-CN+/-
x
F+/-CN+/-
x
F+/+CN+/-
F+/-CN+/+
N = NFATC1
C = SCL-HSC-CreER
F = FLT3ITD
F+/+CN+/+

Supplement: Supplementary file 1 — Figure S1. Breeding scheme. The genotypes used for the experiments are marked with red boxes: FLT3ITD homozygous (F+/+), SCL-HSC-CreER:NFATC1 homozygous (CN+/+), and FLT3ITD:SCL-HSC-CreER:NFATC1 homozygous (F+/+CN+/+). Note: in all the SCL-HSC-CreER–baring animals, the zygosity of the Cre allele is unknown. (PPTX 44 kb) [file 13045_2019_765_MOESM1_ESM.pptx]

## Slide 1
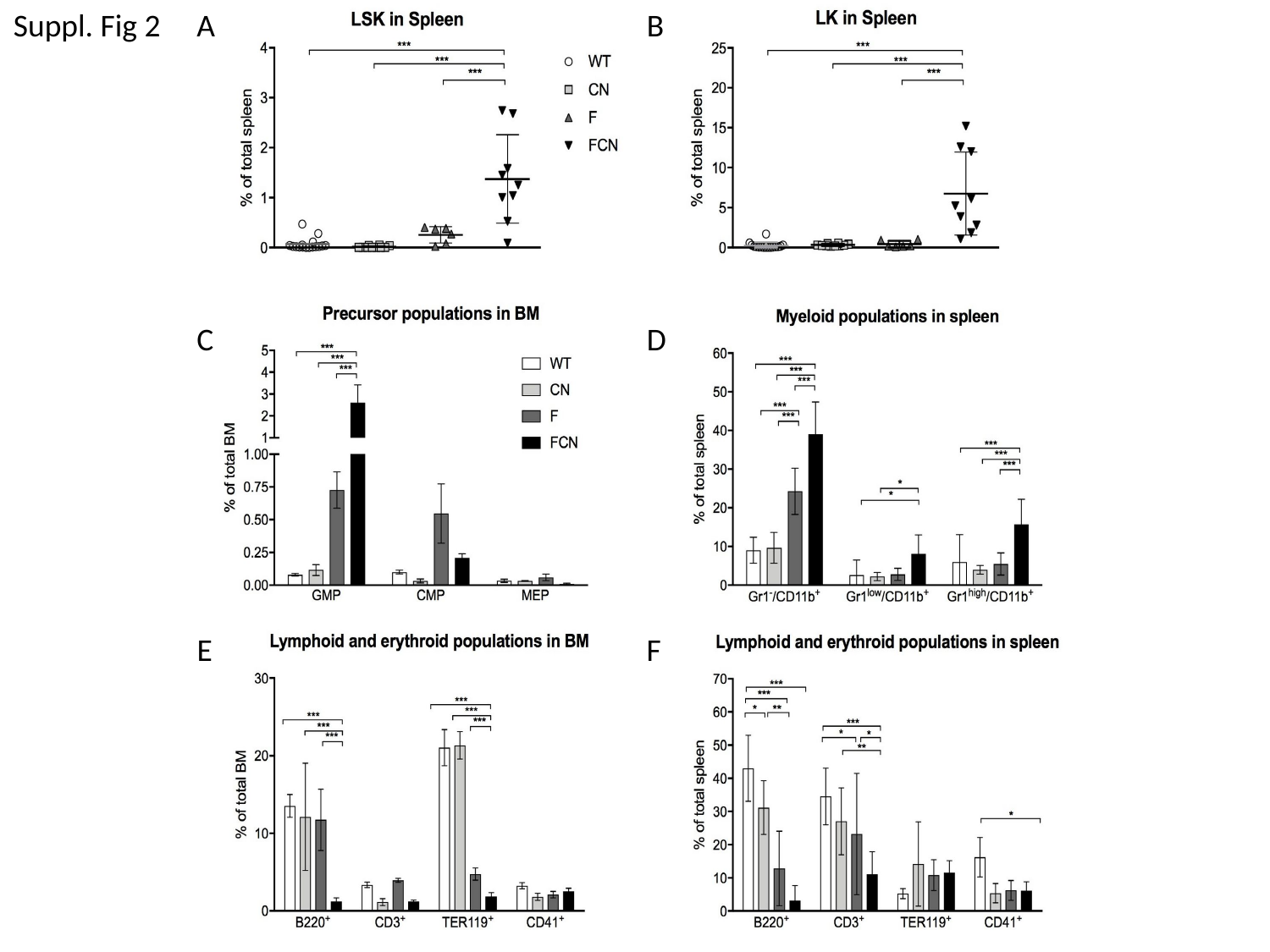

Suppl. Fig 2
A
B
C
D
E
F

Supplement: Supplementary file 2 — Figure S2. Immunophenotyping of bone marrow and spleen. (A) Relative frequencies of LSK (mean ± SD. n = 6 to 16). (B) Relative frequencies of LK (mean ± SD. n = 6 to 16). (C) Relative frequencies of GMP, CMP, and MEP (mean ± SEM. n = 3 to 4). (D) Relative myeloid populations (mean ± SD. n = 6 to 16). (E) Relative frequencies of B-cells (B220+), T-cells (CD3+), erythrocytes (TER119+) and megakaryocytes (CD41+) (mean ± SEM. n = 3 to 10). (F) Relative frequencies of B-cells (B220+), T-cells (CD3+), erythrocytes (TER119+) and megakaryocytes (CD41+) (mean ± SEM. n = 3 to 10). A 1-way ANOVA or 2-way ANOVA with Tukey’s multiple comparison was used for p values. *p < 0.05, **p < 0.01, ***p < 0.001. (PPTX 1112 kb) [file 13045_2019_765_MOESM2_ESM.pptx]

## Slide 1
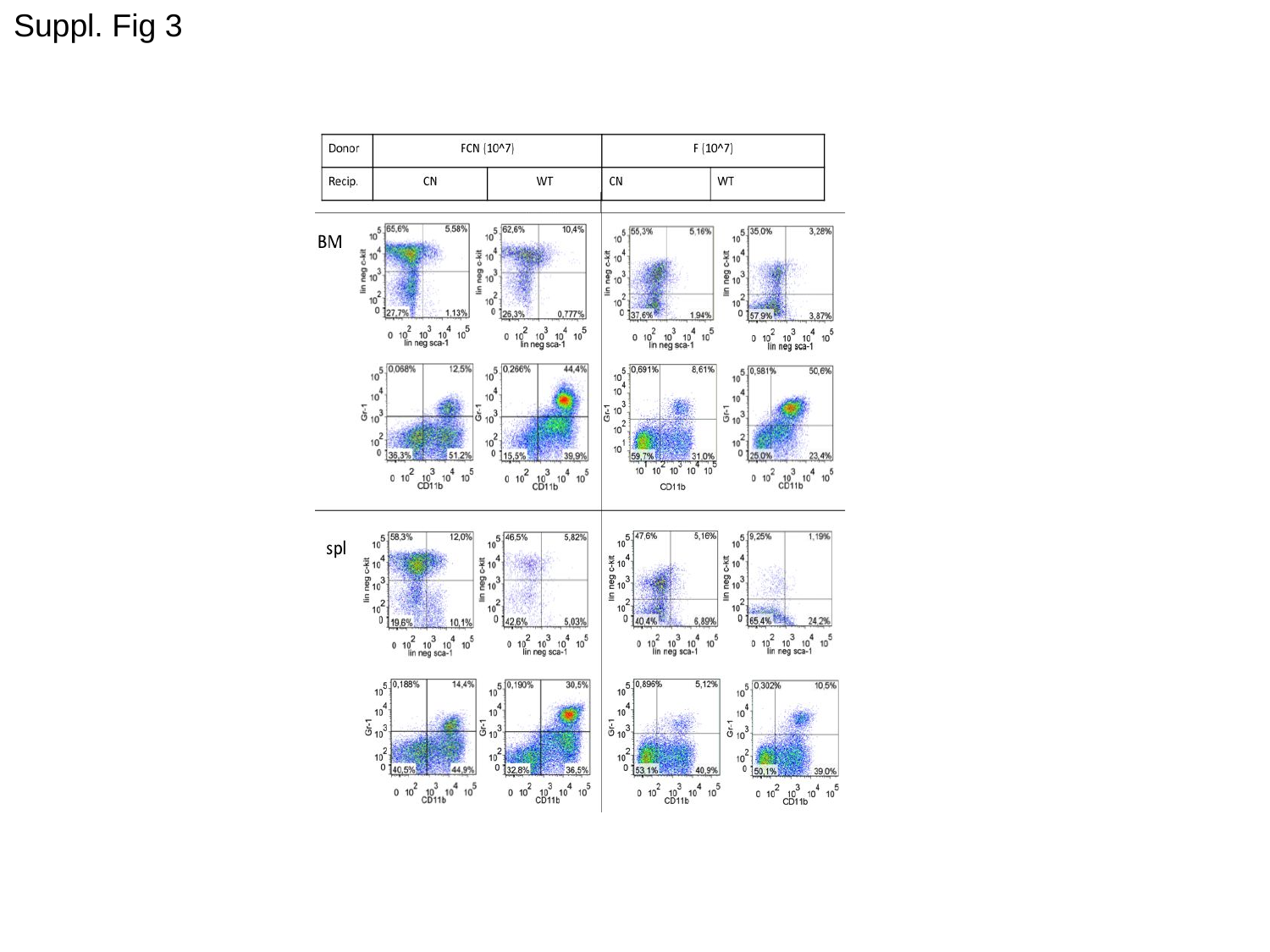

Suppl. Fig 3

Supplement: Supplementary file 3 — Figure S3. Immunophenotyping of bone marrow and spleen after transplantation into CN or WT recipients. 107 total bone marrow cells from F or FCN donor mice were transplanted into irradiated WT recipients. FACS analysis of LSK/LK and myeloid populations are shown for each recipient mouse. (PPTX 484 kb) [file 13045_2019_765_MOESM3_ESM.pptx]
