# Supplementary material for: Nuclear factor of activated T-cells, NFATC1, governs FLT3ITD-driven hematopoietic stem cell transformation and a poor prognosis in AML
Source: J Hematol Oncol. 2019 Jul 8;12:72. doi: 10.1186/s13045-019-0765-y (PMC6615262; doi:10.1186/s13045-019-0765-y)
Supplement: Supplementary file 4 — Figure S4. GSEA analysis from C3 H database for mutual FCN and F genes. Summary table for analysis from GSEA H database: 14 pathways enriched in both FCN and F (only values for FCN are shown). (PPTX 80 kb) [file 13045_2019_765_MOESM4_ESM.pptx]

## Slide 1
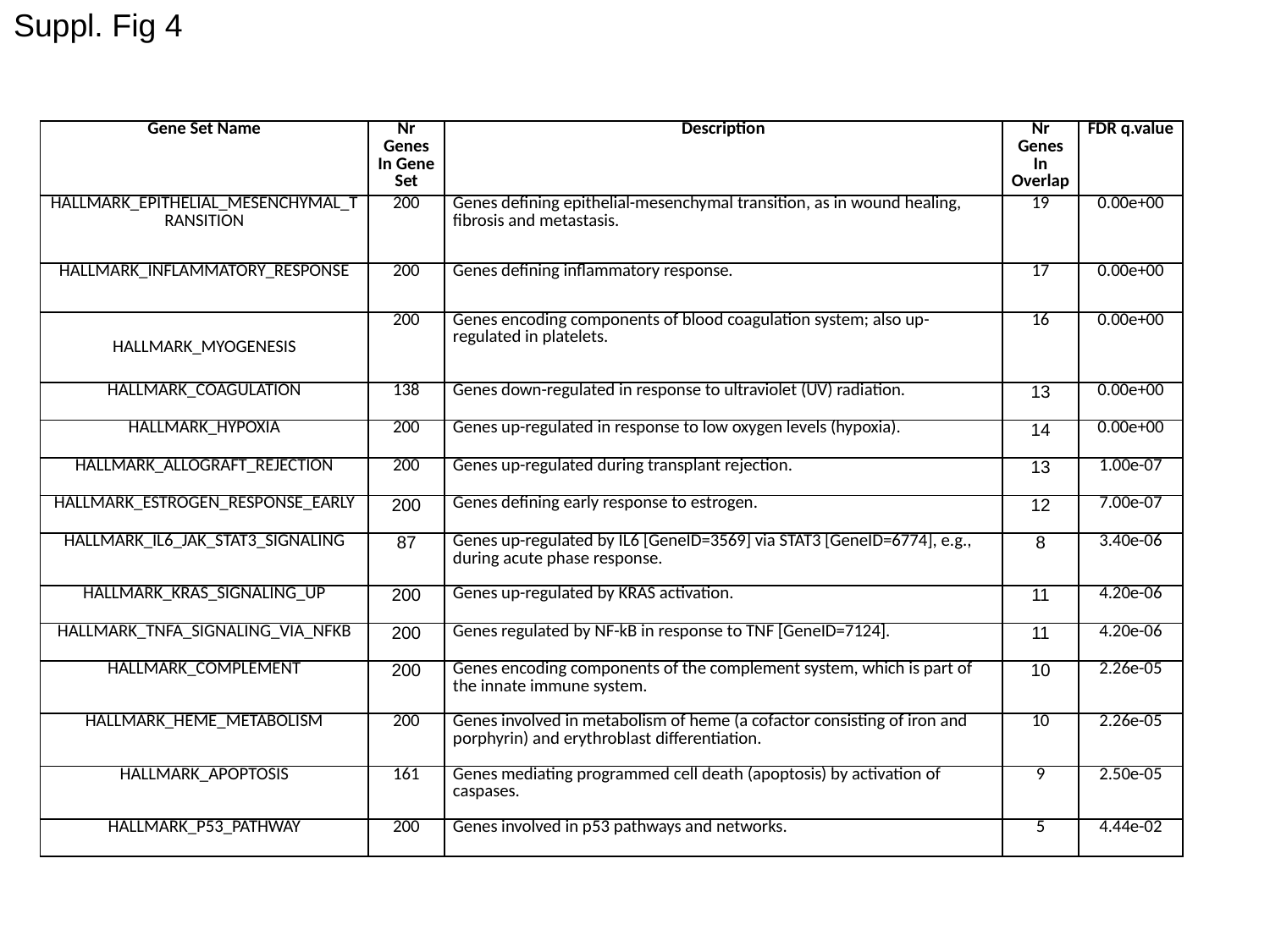

Suppl. Fig 4
| Gene Set Name | Nr Genes In Gene Set | Description | Nr Genes In Overlap | FDR q.value |
| --- | --- | --- | --- | --- |
| HALLMARK\_EPITHELIAL\_MESENCHYMAL\_TRANSITION | 200 | Genes defining epithelial-mesenchymal transition, as in wound healing, fibrosis and metastasis. | 19 | 0.00e+00 |
| HALLMARK\_INFLAMMATORY\_RESPONSE | 200 | Genes defining inflammatory response. | 17 | 0.00e+00 |
| HALLMARK\_MYOGENESIS | 200 | Genes encoding components of blood coagulation system; also up-regulated in platelets. | 16 | 0.00e+00 |
| HALLMARK\_COAGULATION | 138 | Genes down-regulated in response to ultraviolet (UV) radiation. | 13 | 0.00e+00 |
| HALLMARK\_HYPOXIA | 200 | Genes up-regulated in response to low oxygen levels (hypoxia). | 14 | 0.00e+00 |
| HALLMARK\_ALLOGRAFT\_REJECTION | 200 | Genes up-regulated during transplant rejection. | 13 | 1.00e-07 |
| HALLMARK\_ESTROGEN\_RESPONSE\_EARLY | 200 | Genes defining early response to estrogen. | 12 | 7.00e-07 |
| HALLMARK\_IL6\_JAK\_STAT3\_SIGNALING | 87 | Genes up-regulated by IL6 [GeneID=3569] via STAT3 [GeneID=6774], e.g., during acute phase response. | 8 | 3.40e-06 |
| HALLMARK\_KRAS\_SIGNALING\_UP | 200 | Genes up-regulated by KRAS activation. | 11 | 4.20e-06 |
| HALLMARK\_TNFA\_SIGNALING\_VIA\_NFKB | 200 | Genes regulated by NF-kB in response to TNF [GeneID=7124]. | 11 | 4.20e-06 |
| HALLMARK\_COMPLEMENT | 200 | Genes encoding components of the complement system, which is part of the innate immune system. | 10 | 2.26e-05 |
| HALLMARK\_HEME\_METABOLISM | 200 | Genes involved in metabolism of heme (a cofactor consisting of iron and porphyrin) and erythroblast differentiation. | 10 | 2.26e-05 |
| HALLMARK\_APOPTOSIS | 161 | Genes mediating programmed cell death (apoptosis) by activation of caspases. | 9 | 2.50e-05 |
| HALLMARK\_P53\_PATHWAY | 200 | Genes involved in p53 pathways and networks. | 5 | 4.44e-02 |
