# Supplementary material for: Nuclear factor of activated T-cells, NFATC1, governs FLT3ITD-driven hematopoietic stem cell transformation and a poor prognosis in AML
Source: J Hematol Oncol. 2019 Jul 8;12:72. doi: 10.1186/s13045-019-0765-y (PMC6615262; doi:10.1186/s13045-019-0765-y)
Supplement: Supplementary file 5 — Figure S5. Working model. Activation of NFATC1 by an inflammatory environment rewires the transcriptional program induced by FLT3-ITD in stem cells, which promotes leukemic transformation. The FLT3-ITD/NFATC1 synergism activates multiple oncogenic pathways such as WNT/B-Catenin, Hedgehog, and K-RAS, causing extensive proliferation and drug resistance. (PPTX 91 kb) [file 13045_2019_765_MOESM5_ESM.pptx]

## Slide 1
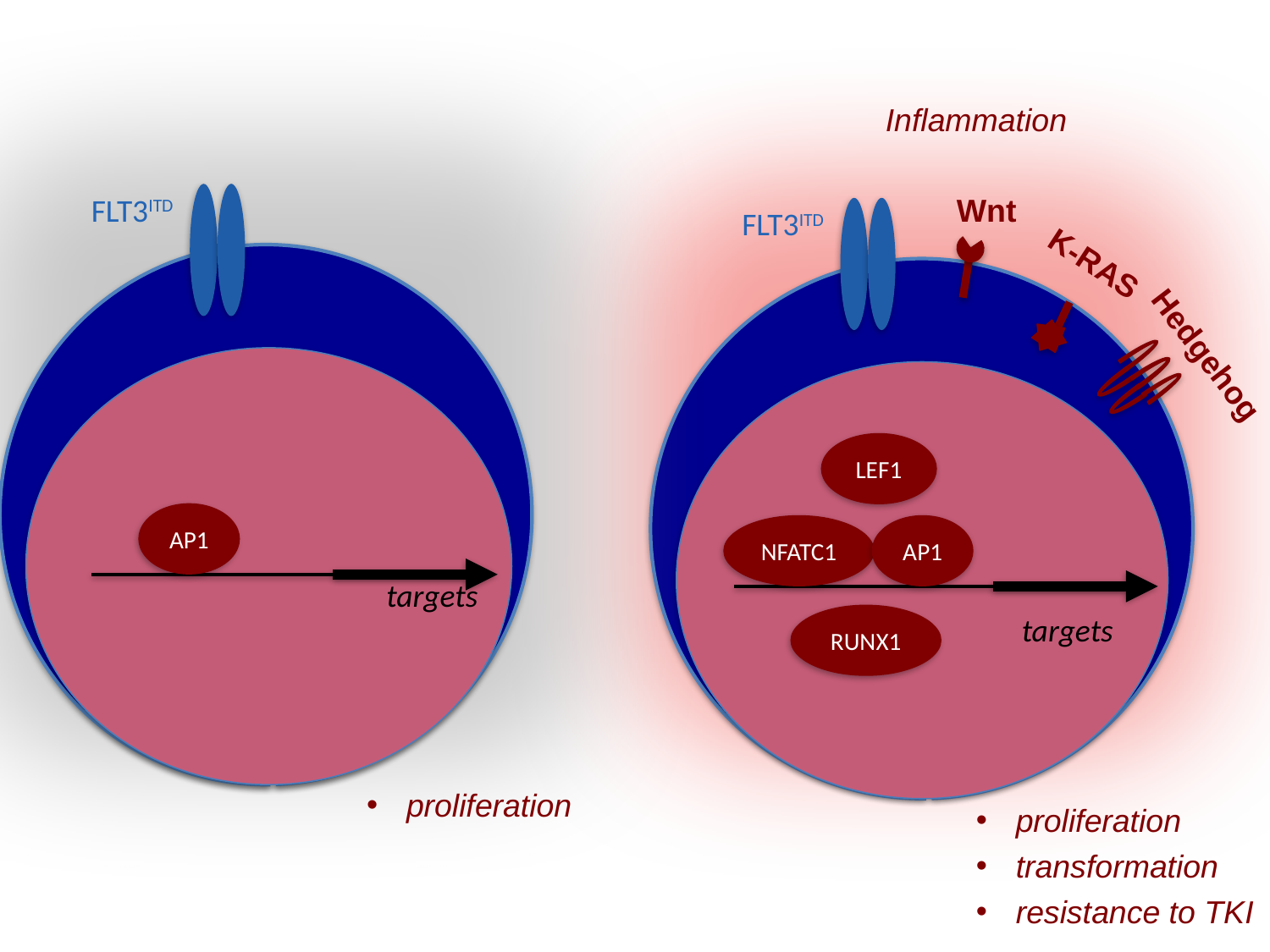

Inflammation
FLT3ITD
Wnt
FLT3ITD
K-RAS
Hedgehog
NFATC1
LEF1
AP1
AP1
targets
targets
RUNX1
proliferation
proliferation
transformation
resistance to TKI
